# Homologous Prime-Boost Vaccination with OVA Entrapped in Self-Adjuvanting Archaeosomes Induces High Numbers of OVA-Specific CD8^+^ T Cells that Protect Against Subcutaneous B16-OVA Melanoma

**DOI:** 10.3390/vaccines4040044

**Published:** 2016-11-17

**Authors:** Felicity C. Stark, Michael J. McCluskie, Lakshmi Krishnan

**Affiliations:** Human Health Therapeutics, National Research Council of Canada, 1200 Montreal Rd., Ottawa, ON K1A 0R6, Canada; Felicity.Stark@nrc-cnrc.gc.ca (F.C.S.); Michael.McCluskie@nrc-cnrc.gc.ca (M.J.M.)

**Keywords:** CD8 T cell response, archaeosome, prime-boost, B16, liposome, tumor vaccine, central memory, effector memory

## Abstract

Homologous prime-boost vaccinations with live vectors typically fail to induce repeated strong CD8^+^ T cell responses due to the induction of anti-vector immunity, highlighting the need for alternative delivery vehicles. The unique ether lipids of archaea may be constituted into liposomes, archaeosomes, which do not induce anti-carrier responses, making them an ideal candidate for use in repeat vaccination systems. Herein, we evaluated in mice the maximum threshold of antigen-specific CD8^+^ T cell responses that may be induced by multiple homologous immunizations with ovalbumin (OVA) entrapped in archaeosomes derived from the ether glycerolipids of the archaeon *Methanobrevibacter smithii* (MS-OVA). Up to three immunizations with MS-OVA administered in optimized intervals (to allow for sufficient resting of the primed cells prior to boosting), induced a potent anti-OVA CD8^+^ T cell response of up to 45% of all circulating CD8^+^ T cells. Additional MS-OVA injections did not add any further benefit in increasing the memory of CD8^+^ T cell frequency. In contrast, OVA expressed by *Listeria monocytogenes* (LM-OVA), an intracellular bacterial vector failed to evoke a boosting effect after the second injection, resulting in significantly reduced antigen-specific CD8^+^ T cell frequencies. Furthermore, repeated vaccination with MS-OVA skewed the response increasingly towards an effector memory (CD62_low_) phenotype. Vaccinated animals were challenged with B16-OVA at late time points after vaccination (+7 months) and were afforded protection compared to control. Therefore, archaeosomes constituted a robust particulate delivery system to unravel the kinetics of CD8^+^ T cell response induction and memory maintenance and constitute an efficient vaccination regimen optimized for tumor protection.

## 1. Introduction

CD8^+^ T cells are an important component of successful anti-tumor immune responses and, therefore, predictably, most cancer vaccines aim to expand tumor specific CD8^+^ T cells. Tumors are adept at masking their presence and can escape recognition and killing by CD8^+^ T cells. Therefore, potent adjuvants that trigger macrophage activation, major histocompatibility complex (MHC) and co-stimulatory molecule expression and modulate the immune check-point responses are thought to be required in an effective anti-cancer vaccine.

Many vaccination strategies have incorporated the use of live vectors such as poxviruses [1,2,3,4], *Salmonella typhimurium* [5,6,7,8,9,10] and *Listeria monocytogenes* [6,8,11] that have the ability to both self-adjuvant and to be genetically-modified to express target antigen. While live attenuated vectors represent a robust method to provide adjuvanting signals, their use can be complicated by the risk of reversion to virulence and anti-vector immunity. Other methods include the use of conventional liposomes derived from eubacterial or synthetic ester phospholipids; these are typically designed to deliver cargo antigen [12], however, they fail to produce strong co-stimulation and must incorporate immuno-stimulants such as Lipid A [13], CpG oligonucleotides [14], or other pathogen-associated molecular patterns (PAMPS) [15] to cause sufficient co-stimulation signals to generate a robust CD8^+^ T cell response.

Archaeosomes are liposome vesicles composed of the polar lipids unique to the domain Archaea. Archaeal lipids bear unique structural signatures with phytanyl fully saturated core lipid tails, ether linked to the glycerol back-bone, unlike ester linked fatty acyl chains of eubacteria [16]. Initially reported by Sprott et al., [17,18,19,20] archaeosomes were found to elicit superior humoral responses towards entrapped cargo (BSA or cholera toxin B subunit) when compared to conventional liposomes. Notably, the antibody responses were equivalent to those achieved with complete Freund′s adjuvant. Many different archaeosomes composed of total polar lipids (TPL) of various genera have since been characterized and the lipids derived from *Methanobrevibacter smithii* (MS) were selected for their lipid composition that optimally elicited profound CD8^+^ T cell effector and memory responses [17,21]. Briefly, MS lipids uniquely comprise of a mixture of archaeols and caldarchaeols (3:2) and are high in phosphoserine (PS) head groups [18]. Caldarchaeols impart membrane rigidity due to their membrane spanning C-40 backbone leading to stable liposomes that can impart prolonged antigen presentation and immune memory [22]. The presence of PS headgroups allows for receptor mediated endocytosis via the PS receptor on antigen presenting cells (APCs) [23]; this is turn facilitates the fusion of the archaeosome membrane with phagosomes, thereby delivering entrapped antigen to MHC Class I processing machinery [23] making them potent inducers of CD8^+^ T cell immunity.

Archaeosomes composed of total polar [17,24] or semi-synthetic [25] archaeal lipid vesicles represent a robust method for inducing tumor protective CTL responses as they can recruit and activate DCs in vivo in mouse models, and deliver cargo antigen to the MHC class I processing machinery causing CD8^+^ T cell activation [22]. Thus, archaeosomes constitute a convenient antigen delivery system with innate adjuvant properties that induce strong co-stimulation and CD8^+^ T cell activation and have also been shown to break tolerance to cancer self-antigens [24].

A fundamental immunological question in the context of cancer immunotherapy that can be addressed with archaeosomes is: what is the maximal threshold of antigen-specific CD8^+^ T cells that can be evoked by vaccination? Archaeosomes are ideal for this inquiry as they are a non-antigenic, non-replicating particulate vaccine delivery system whose efficacy may not be compromised with repeated boosting due to circulating vesicle-specific neutralizing antibodies. Overall this study was aimed at evaluating the potency of a nanoparticle delivery system (archaeosomes) to induce a CD8^+^ T cell response in a repeat dose setting. Ovalbumin was chosen as a model antigen as it has established methods in place to track OVA-specific T cell responses. Furthermore, it is a xenogenic antigen and is not subject to the host tolerance factors that would hinder self-antigens; thus, making it preferable for addressing questions of maximal threshold of antigen-specific CD8 T cell responses. In choosing a vaccine model to compare with archaeosomes, we selected a live vaccine *Listeria monocytogenes* expressing OVA (LM-OVA). LM-OVA has a proven track record of eliciting rapid CD8^+^ T cell responses with long lasting memory, and is also capable of providing long lasting protection from tumor challenge [26]. Thus, the response to the same highly immunogenic antigen expressed in the context of two contrasting antigen delivery systems could be compared.

We demonstrate that a vaccination regimen with archaeosomes can be optimized to achieve up to ~45% OVA-specific CD8^+^ T cells with just three repeat doses. Furthermore, the vaccination schedule influences the proportion of effector versus central memory phenotype cells. Finally, the proportion and phenotype of antigen-specific CD8^+^ T cell response correlates to tumor protection.

## 2. Materials and Methods

### 2.1. Vaccine Delivery Systems and Route of Immunization

Archaeosomes were prepared from the total polar lipids of *Methanobrevibacter smithii* as described previously [27]. Briefly, the model protein OVA, type VI (Sigma-Aldrich, Oakville, ON, Canada) was encapsulated within archaeosomes by hydrating dried total polar lipids. Vesicle diameter was reduced to ~100 nm by sonication and assessed with a particle sizer (Nicomp 350, Santa Barbara, CA, USA). Non-entrapped OVA was removed from solution by ultracentrifugation at 327,000× *g*. The supernatant was discarded and the pellet was re-suspended in 1–2 mL of water by gentle vortexing and filtered manually through a 0.45 μm, 25 mm diameter syringe driven sterilizing filter. Encapsulated OVA was quantified by SDS-PAGE and the antigen: lipid ratio was found to be in the range of 20 µg OVA in 0.3–0.7 mg of archael lipids. Working stocks for vaccines were diluted in PBS (20 µg OVA/100 µL PBS).

Recombinant *L. monocytogenes* (LM-OVA) was generated and grown as previously described and contains the chromosomally-integrated truncated OVA gene (aa 134–387) [28,29], including the CD8^+^ T cell epitope SIINFEKL (257–264) as well as the CD4^+^ T cell epitope ISQAVHAAHAEINEAGR (323–339). Frozen LM-OVA bacterial stocks were thawed and diluted in 0.9% NaCl prior to injection.

Mice were immunized by subcutaneous (s.c.) injection (20 µg OVA entrapped in archaeosomes or 1000 CFU LM-OVA in 100 μL saline) in the mid dorsal flank. Since LM-OVA is a replicating vaccine, equalizing the amount of antigen expressed and delivered in vivo to that of archaeosomal delivery was not possible. Rather, the dose for each vaccine was chosen to optimally induce similar frequencies (~10%) of OVA-CD8^+^ T cells on day 28, after a prime-boost vaccination regimen at day 0 and 21. Numbers of animals per group and vaccination regimen are listed in the figure legends.

### 2.2. Mouse Strains

Six-to-eight week old female C56BL/6 mice were obtained from The Jackson Laboratory (Bar Harbor, ME, USA). Mice were maintained at the small animal facility of the National Research Council Canada (NRC) in accordance with the guidelines of the Canadian Council on Animal Care. All animals use protocols were approved by the NRC Animal Care Committee (Protocol 2011.24).

### 2.3. Tumor Model (B16-OVA, Melanoma)

B16F0-OVA (expressing plasmid derived full length OVA) cells were obtained from Dr. Edith Lord (University of Rochester, Rochester, New York, NY, USA) and cultured as described previously [10,30]. Solid tumors were induced with s.c. injection of 1 × 10^6^ B16-OVA cells. From day five onwards, detectable solid tumor was measured using Digimatic Digital calipers (Mitutoyo 500-196, Aurora, IL, USA). Tumor size, expressed in mm^2^, was obtained by multiplication of diametrically perpendicular measurements. Animals were monitored for long-term survival. However, in order to minimize pain and discomfort, mice were euthanized when tumors reached 300 mm^2^.

### 2.4. Assessment of In Vivo Cytolytic Activity

In vivo cytolytic activity of CD8^+^ T cells was enumerated as described previously [31]. Briefly, vaccinated recipient mice were injected with carboxyfluorescein succinimidyl ester (CFSE) stained target cells previously coated with OVA-peptide and non-coated cells as a control. The percentage of in vivo killing was calculated 24 h later based on the survival proportions of target cell populations.

### 2.5. Detection of OVA-Specific CD8^+^ T Cells

At various time points (before vaccination, 1 week after vaccination and at regular intervals afterwards), spleen samples were harvested and blood (50–100 µL) was collected by mandibular bleeding. In a second study (staggered vaccination), blood samples were taken one day before tumor challenge and at days 0, 8, 20, 41, and 58. Spleens were processed by mashing between the frosted ends of two glass slides in RPMI 1640 medium (Invitrogen, Life Technologies, Grand Island, New York, NY, USA) supplemented with 8% fetal bovine serum (FBS) (HyClone Laboratories, Logan, UT, USA) (R8). Splenocytes were passed through 45 μm Falcon cell strainers, washed, and then treated with 500 μL of RBC lysing buffer (Sigma-Aldrich, Oakville, ON, Canada); blood samples were treated with RBC lysing buffer after antibody staining. Cells were washed by centrifugation at 400 g for 8 min and finally re-suspended in 5–10 mL of R8 medium. Live cell number was enumerated by trypan blue exclusion using a hemocytometer.

Spleen samples were first blocked with anti-Fc receptor antibody (anti-CD16) for 5 min at 4 °C. Fresh spleen and blood samples were stained with antibodies against CD8^+^ and CD62L as well as with the MHC tetramer H-2K^b^OVA. All antibodies were obtained from BD Biosciences (Mississauga, ON, Canada). H-2K^b^OVA-tetramer was obtained from Beckman Coulter (Mississauga, ON, Canada). Cells were washed with PBS, fixed with 0.5% paraformaldehyde, and acquired on a BD FACS Canto analyzer (Becton, Dickinson and Company, Franklin Lakes, NJ, USA). FSc. SSc. gating was used to locate lymphocytes, exclude doublets, and exclude debris and dead cells, a further gate was set to identify CD8^+^, MHC tetramer H-2K^b^OVA+ cells. Single stained antibody controls were used to set compensation values between channels to prevent overlapping signals creating false-positives. Additionally, fluorescence minus one (FMO) gating controls were used. Flow cytometry data were analyzed using the FACS Diva^®^ software (Becton, Dickinson and Company, Franklin Lakes, NJ, USA).

## 3. Results

### 3.1. Multiple Boosting with MS-OVA Induced High Numbers of Circulating CD8^+^ T Cells

**Figure 1:**
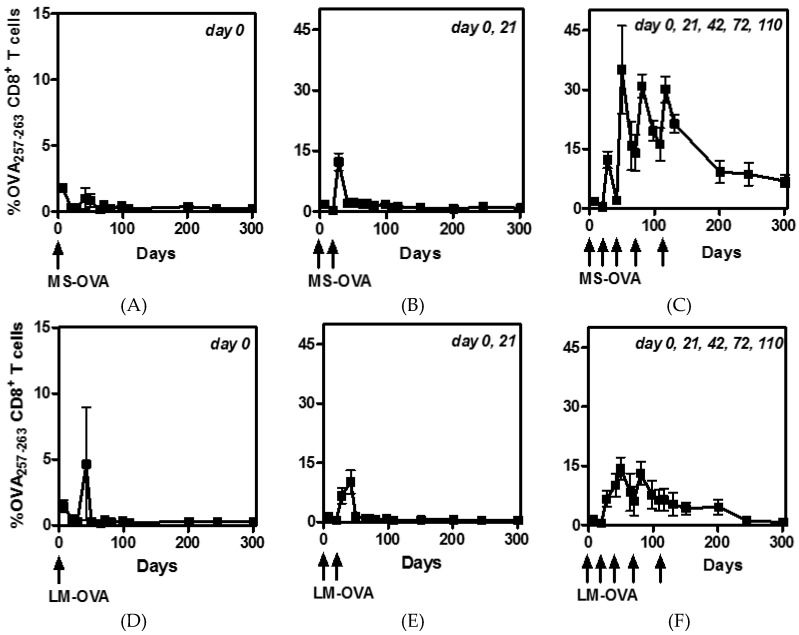
CD8^+^ T cell frequency following prime-boost vaccinations with MS-OVA or LM-OVA. C56BL/6 mice were vaccinated by s.c. injection with 20 µg of MS-OVA (panels (**A**–**C**)) or 10^4^ LM-OVA (panels (**D**–**F**)) in the middle of the dorsal flank either once, twice or five times (day 0, 21, 42, 72, 110) as indicated at the top of each panel. Blood was taken before vaccination, seven days after and 21 days after each vaccination as well as at later time-points. Blood was stained with anti-CD8 antibody as well as OVA_257–263_ tetramer and analyzed by flow cytometry. *n* = 3/time point.

In order to determine the maximum possible CD8^+^ T cell response that can be achieved, MS-OVA or LM-OVA were administered (s.c.) to C57BL/6 mice at specific time intervals either once, twice or five times as indicated in the figure legends. Doses were given at ≥3 week intervals to allow the primed effector CD8^+^ T cells time to rest before being reactivated. The frequency of OVA-specific CD8^+^ T cells was monitored in the blood before vaccination, at seven days after vaccination and then at regular intervals up to 300 days post first dose. A single dose of MS-OVA or LM-OVA elicited equivalently modest responses with only ~2% OVA-specific CD8^+^ T cells in circulation by day seven and ~0.3% OVA-specific CD8^+^ T cells up to 300 days post vaccination (Figure 1A,D). A second dose of MS-OVA or LM-OVA at day 21 resulted in a ≥5-fold increase in OVA-specific CD8^+^ T cells to ~10%–15% by day 28 (Figure 1B,E). The addition of subsequent vaccinations at day 42, 72, and 110, further increased the percent of OVA-specific CD8^+^ T cells with MS-OVA but not LM-OVA (Figure 1C,F). Notably, at the time of the third vaccination (day 42), the frequency of primed OVA-specific CD8^+^ T cells was significantly lower in MS-OVA than LM-OVA vaccinated mice (*p* = 0.04). Thus, a clear disparity in the timing of contraction was observed following MS-OVA or LM-OVA vaccination regimen. Additionally, after the third vaccination with LM-OVA, very little T cell expansion occurred (Figure 1F). In contrast, after the second vaccination with MS-OVA, the OVA-specific CD8^+^ T cells underwent quick contraction, and the third vaccination with MS-OVA evoked a rapid proliferation resulting in more than 30% OVA-specific CD8^+^ T cells (Figure 1C) in the circulation. The fourth and fifth vaccinations also induced very little T cell proliferation with LM-OVA but marked proliferation with MS-OVA. Even at 300 days post initial vaccination, ~5% of OVA-specific CD8^+^ T cells were still detectable in the blood of mice after multiple MS-OVA vaccinations compared to ~1% OVA-specific CD8^+^ T cells after multiple LM-OVA vaccinations (Figure 1C,F).

### 3.2. CD62L Expression on CD8^+^ T Cells Reduces with Multiple Boosting

**Figure 2:**
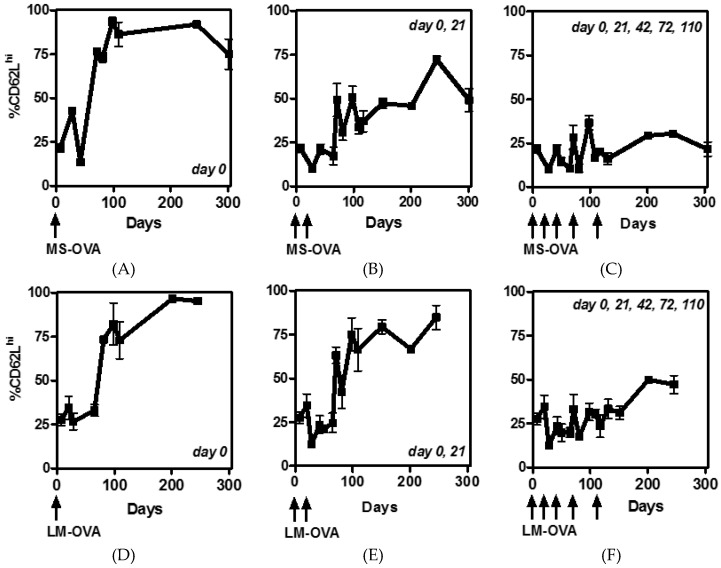
CD62L^high^ expression on responding CD8^+^ T cells following prime-boost vaccinations with MS-OVA or LM-OVA. C56BL/6 mice were vaccinated s.c. with 20 µg of MS-OVA (panels (**A**–**C**)) or 10^4^ LM-OVA (panels (**D**–**F**)) in the middle of the dorsal flank either once, twice or five times (day 0, 21, 42, 72, 110) as indicated at the top of each panel. Blood samples were taken before vaccination, seven days after and 21 days after each vaccination, as well as at later time-points. Blood was stained with anti-CD8 antibody, OVA_257–263_ tetramer and anti-CD62L antibody and analyzed by flow cytometry. Percent CD62L was obtained from a gated population on OVA_257–263_^+^ CD8^+^ T cells. Mean ± SD, *n* = 3/timepoint.

To further characterize the phenotype of OVA-specific CD8^+^ T cell response elicited by MS-OVA or LM-OVA after single or multiple vaccinations, blood samples were stained for CD62L. While decreased CD62L expression on CD8^+^ T cells is associated with both effector and effector memory phenotypes and an overall increase in cytotoxicity, a predominantly CD62L^high^ CD8^+^ T cell population has been associated with central memory and increased re-call CD8^+^ T cell proliferation and efficacious anti-tumor response [26]. After one vaccination of MS-OVA or LM-OVA causing an initial (day 7) induction of CD62L^low^ effector CD8^+^ T cells, there was a subsequent gradual increase in CD62L^high^ memory cells. A significant amount (~75%) of the OVA-specific CD8^+^ T memory cells were CD62L^high^ by day 70 and remained high for >200 days (Figure 2A,D). In mice that received a booster injection with MS-OVA, only about 50% of OVA-specific CD8^+^ T cells were CD62L^high^ indicating a shift in the overall phenotype of the memory CD8^+^ T cells compared to one vaccination (Figure 2B). In contrast, after boosting with LM-OVA, a large proportion of the cells regained a high expression of CD62L, albeit at slightly lower levels compared to priming alone (Figure 2E). Subsequent boosting with either MS-OVA or LM-OVA reduced the percent CD62L^high^ CD8^+^ T cells to ~25% and ~50%, respectively, even at late time-points (>200 days) (Figure 2C,F).

**Figure A1:**
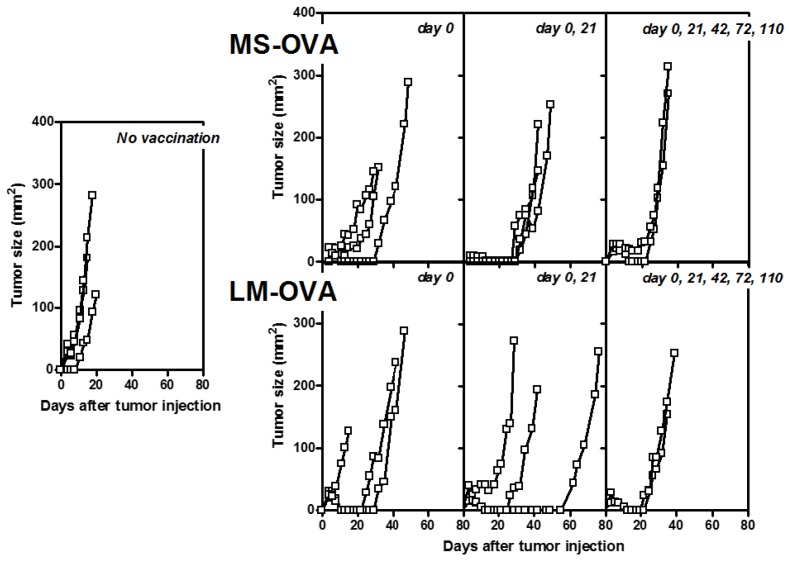
Tumor challenge 323 days following regular prime-boost vaccinations with MS-OVA or LM-OVA. C56BL/6 mice were vaccinated by s.c. injection with 20 µg MS-OVA or 10^4^ of LM-OVA in the middle of the dorsal flank at day 0, 21, 42, 72, and 110 as indicated in the legend. Three hundred twenty-three days after the initial vaccination, 10^6^ B16-OVA cells were injected s.c. in the lower dorsal flank. Survival was measured over time.

**Figure A2:**
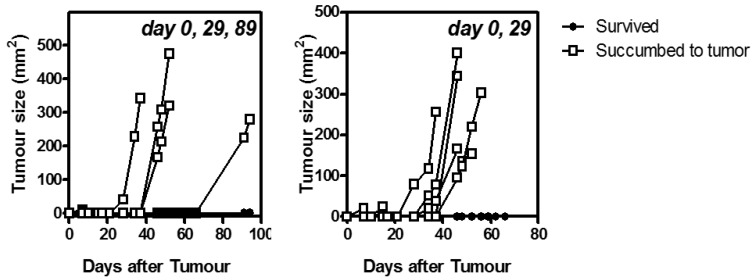
Tumor challenge following staggered prime-boost vaccinations with. MS-OVA. C56BL/6 mice were vaccinated by s.c. injection with 20 µg of MS-OVA in the middle of the dorsal flank at day 0, 29, and 89 as indicated in the legend. Two hundred sixteen days after initial vaccination 10^6^ B16-OVA cells were injected s.c. in the lower dorsal flank. Tumor volume is measured over time. *n* = 3–7.

Overall, while boosting with MS-OVA significantly increases the frequency of OVA-specific CD8^+^ T cells, a marked decrease in CD62L^high^ T_CM_ cells was observed at later time-points. In the case of LM-OVA, multiple boosting did not augment an increase in the frequency of OVA-specific CD8^+^ T cells past the second dose, however there was a discernable decrease in the frequency of CD62L^high^ cells (relative to one or two LM-OVA injections) although the overall frequency of CD62L^high^ cells was less than that observed for MS-OVA vaccinated mice. Three hundred twenty-two days after the initial vaccination, all mice were challenged with a s.c. injection of 10^6^ B16-OVA cells. Interestingly, most vaccinated mice were afforded some protection from tumor development, but there was no significant difference in protection between MS-OVA and LM-OVA vaccinated groups (Appendix Figure A1 and Figure A2).

### 3.3. Staggered Boosting Results in a High Frequency of OVA-CD8^+^ T Cells and Long-Term Tumor Protection

**Figure 3:**
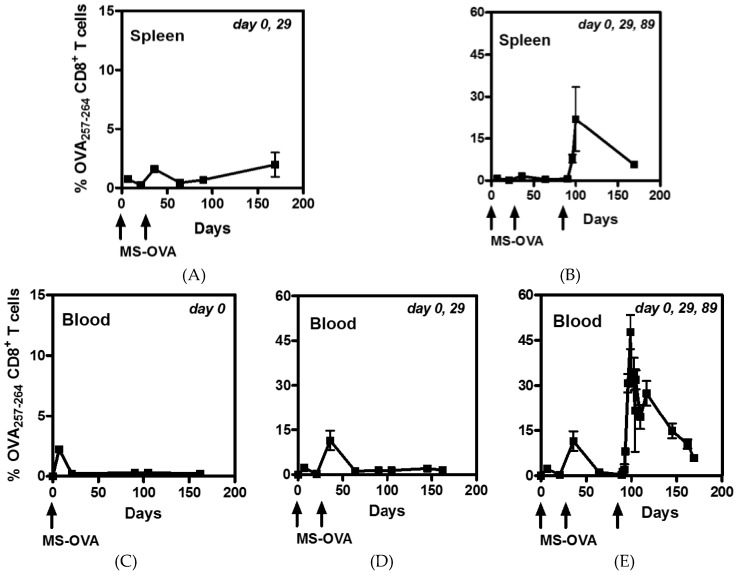
CD8^+^ T cell frequency following staggered prime-boost vaccinations with MS-OVA. C56BL/6 mice were vaccinated by s.c. injection with 20 µg of MS-OVA in the middle of the dorsal flank at day 0, 29, and 89 as indicated at the top of each panel. (**A**,**B**) Spleen (*n* = 2/time point) and (**C**–**E**) blood (*n* = 3–5/time point) were collected before vaccination, seven days after and 21 days after each vaccination, as well as at later time-points. The blood was stained with anti-CD8 antibody as well as OVA_257–263_ tetramer and analyzed by flow cytometry.

The re-exposure of CD8^+^ T cells to antigen at short intervals can lessen their proliferation potential and lead to activation induced cell death due to overstimulation [32]. While it is currently not known how much of a delay is suitable to maximize the CD8^+^ T cell response, in an effort to improve the OVA-specific CD8^+^ T cell response, we increased the time intervals between boosts such that the second and third vaccinations were given on days 29 and 89. The frequency of OVA-specific CD8^+^ T cells was measured in the spleens of mice at various time-points following two (Figure 3A) or three (Figure 3B) vaccinations with MS-OVA and in the blood from mice vaccinated once (Figure 3C), twice (Figure 3D), or three times (Figure 3E) with MS-OVA. Remarkably, using the extended schedule (0, 29, and 89 days), up to 50% of CD8^+^ cells in the blood were found to be specific for OVA 10 days after the third vaccination (Figure 3E). Mice were challenged with B16 OVA tumor cells on day 216 and their survival monitored.

**Figure 4:**
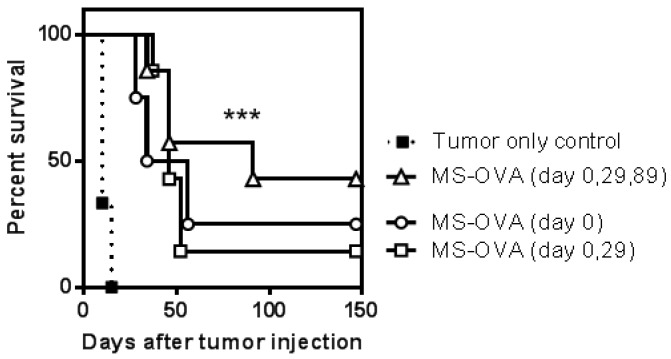
Tumor challenge following staggered prime-boost vaccinations with MS-OVA. C56BL/6 mice were vaccinated by s.c. injection with 20 µg of MS-OVA in the middle of the dorsal flank at day 0, 29, and 89 as indicated in the legend. Two hundred sixteen days after initial vaccination 10^6^ B16-OVA cells were injected s.c. in the lower dorsal flank. Survival was measured over time. *** All vaccinated animals survived significantly longer than control *p* < 0.0001, log-rank (Mantel-Cox) Test. *n* = 3–7.

Mice vaccinated three times had a greater median survival (91 days) than mice vaccinated once or twice (46 days), however the difference was not statistically significant. Overall, vaccinated animals survived significantly longer (log-rank, Mantel-Cox, *p* < 0.0001) than control mice, which had a median survival of 10 days (Figure 4).

**Figure 5:**
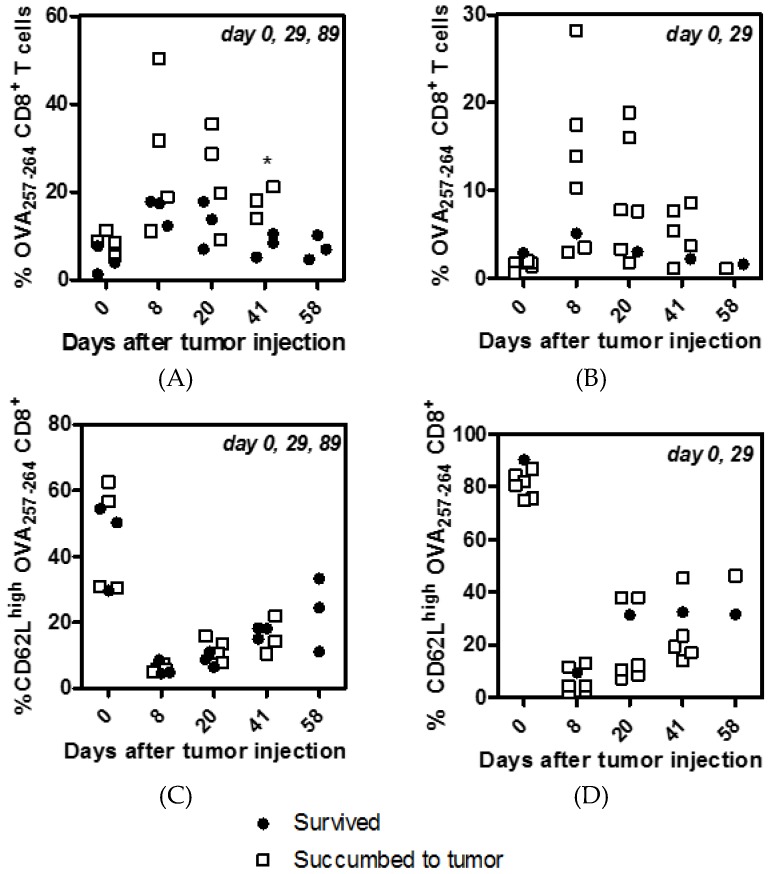
CD8^+^ T cell frequency and CD62L phenotype following tumor challenge after vaccination. C56BL/6 mice were vaccinated by s.c. injection with MS-OVA in the middle of the dorsal flank at days 0, 29 and 89 (panels **A** and **C**) or 0, 29 (panels **B** and **D**) as indicated at the top of each panel. Two-hundred sixteen days after initial vaccination, 10^6^ B16-OVA cells were injected s.c. in the lower dorsal flank. Blood samples were taken after tumor injection and assayed for the frequency of OVA_257–264_ specific CD8^+^ T cells (panels **A** and **C**) and their percent expression of CD62L (panels **B** and **D**). Surviving mice and those that succumbed to tumor mice were segregated for analysis. (**A**) Two-tailed *t*-test with Welch′s correction for unequal variance *, *p* = 0.02. Data are graphed for individual mice, *n* = 3–7/time point.

Blood samples were taken at various time-points (0, 8, 20, 41, and 58 days) after tumor cell injection and the frequency of OVA-specific CD8^+^ T cells was plotted for mice receiving three (Figure 5A) or two (Figure 5B) vaccinations. Mice were segregated into two groups to show those that subsequently survived tumor challenge past 70 days (closed circle) or those that succumbed to tumors (open square). Frequency (%) of CD62L^high^ OVA-specific CD8^+^ T cells were also shown for mice receiving three (Figure 5C) or two (Figure 5D) vaccinations. Blood samples taken immediately before tumor challenge (day 0) show a CD62L^high^ phenotype for the majority of OVA- specific CD8^+^ T cells; this was more prominent in mice vaccinated twice (Figure 5D) compared to mice vaccinated three times (Figure 5C) (***, *p* < 0.001). This indicates that mice given a third vaccination had a more heterogenous CD62L^low^ and CD62L^high^ OVA-specific CD8^+^ T cell population than mice which received only two vaccinations. Eight days after tumor injection the blood profile indicated that the majority of cells obtained had a CD62L^low^ effector T cell (T_E_) phenotype (Figure 5C,D) and a substantial expansion of OVA-specific CD8^+^ T cells was evident (Figure 5A,B). Notably, the expansion of OVA-specific CD8^+^ T cells was more prominent in those mice that eventually succumbed to tumor burden, this could be as a result of the larger tumor and associated increase in antigen causing re-stimulation of OVA-specific CD8^+^ T cells. In summary, we observed that >100 days after vaccination, prior to tumor challenge, mice vaccinated twice have significantly less blood resident OVA-specific CD8^+^ T cells (**, *p* < 0.01) (Figure 5A,B) but they expressed significantly more CD62L (***, *p* < 0.001) (Figure 5C,D) compared to mice vaccinated three times yet they had similar protection from a tumor challenge. This indicates that while a T_CM_ phenotype (CD62L^high^) is generally considered desirable for an anti-tumor response [32], the presence of a larger number of CD8^+^ T cells with an effector function (T_EM_, CD62L^low^) may compensate, leading to equivalent tumor protection, at least in this tumor model.

## 4. Discussion

A central challenge for vaccinologists is to create a CD8^+^ T cell response with maximal potency. By utilizing archaeosome adjuvants, we queried whether CD8^+^ T cell numbers could be continually increased with repeated dose vaccines.

MS-OVA is a unique delivery system that elicits no known anti-carrier immune responses and is capable of homologously boosting OVA-specific CD8^+^ T cell responses substantially more than a second injection of LM-OVA, a potent primary CD8^+^ T cell inducer [22]. Therefore, in a prophylactic model, we repeatedly vaccinated mice with MS-OVA to induce a maximal frequency of OVA-specific CD8^+^ T cells and compared the responses with multiple LM-OVA doses. Priming and boosting with LM-OVA (at 21 day intervals) expanded OVA-specific CD8^+^ T cell responses to ~15% of all circulating CD8^+^ T cells (Figure 1). Comparatively, priming and boosting with MS-OVA expanded circulating OVA-specific CD8^+^ T cells frequency to ~35%, seven days following the third vaccination. Notably, a fourth and fifth vaccination did not further increase T cell responses past ~35%. The factors that limit continuous T cell expansion may include T cell suppressive mechanisms including the expression of checkpoint inhibitors such as PD-1, LAG-3, CTLA-4, Tim3, or activation of regulatory T cells [33]. Alternately, there may be a maximum quantitative threshold of CD8^+^ T cell activation as they compete for interactions with APCs and cytokine growth factors for proliferation. It is also possible that later vaccinations were introduced too early; after each immunization the length of time it takes to regain CD62L expression increases (Figure 2); T cells with a decreased level of cell surface CD62L have decreased in proliferative potential than their counterparts. Therefore, delaying the booster shot could allow for the generation of T cells with a greater proliferative potential, further expanding the OVA-specific CD8^+^ T cell frequency.

In order to address this, the booster shots were staggered in a second study and additional sample collection points added. After confirmation of the up-regulation of CD62L on OVA-specific CD8^+^ T cells, MS-OVA vaccinations were given at day 0, 29, and 89 (Figure 3). Antigen specific CD8^+^ cell frequencies were detectable at a similar frequency of 30%, seven days after the third vaccination, as was obtained following vaccination at 0, 21, and 42 days. However an additional time point taken 10 days after the third vaccination showed a very high frequency of OVA-specific CD8^+^ T cells of 40%–58%. In a study of cluster vaccinations with OVA protein and poly(I:C) by Wick et al. [34], daily vaccinations induced a maximum of 30% antigen specific cells in the periphery by day seven after cluster vaccination similar to our regular interval vaccination model (0, 21, 42, 72, and 110). They report that the response declined to 15% by day 10 and did not achieve the high frequency observed in our staggered vaccination model of 40%–58% after the third vaccination (0, 21, 89) [34]. It is possible that a maximum threshold for CD8^+^ T cell activation is an evolutionary host adaptation that ensures tissue homeostasis and readiness to respond to multiple pathogens at all times. Nevertheless, archaeosomes appear to be able to induce a remarkably high level of antigen specific CD8^+^ T cells.

In addition to generating a higher number of tumor specific CD8^+^ T cells, the memory phenotype is thought to be important for the overall anti-tumor immune response [26]. Previously, we have shown that when memory OVA-specific CD8^+^ T cell frequencies are similar and CD62L expression is low or their ligand is absent, long lasting tumor protection is impaired [26,35]. At late time points (>200 days) after a single dose of MS-OVA or LM-OVA, a low frequency of OVA-specific CD8^+^ T cells remain (~0.1%) and they are predominantly CD62L^high^. Conversely, multiple doses of MS-OVA or LM-OVA induce higher frequencies of OVA-specific CD8^+^ T cells that are predominantly CD62L^low^. Nevertheless, vaccinated mice receiving single or multiple vaccine doses had similar protection when tumor challenge occurred 323 days post initial vaccination (Appendix Figure A1). This highlights the potency of 0.1% circulating OVA-specific CD8^+^ T_CM_ cells. It is possible that aging of the mice could have been a confounding factor that lessened T cell responses, as older mice are known to have increased numbers of T_regs_ and myeloid suppressor cells [36,37,38,39,40]. Nonetheless, a single vaccination with LM-OVA or MS-OVA was capable of providing some protection from tumors at this late challenge date, similar to what was achieved with multiple vaccinations that yield a higher frequency of circulating OVA-specific CD8^+^ T cells. This reinforces the idea that even a low frequency of tumor reactive CD8^+^ T cells of the T_CM_ phenotype can provide protection from tumor. In a separate experiment, mice were vaccinated with MS-OVA at staggered intervals and tumor challenge was introduced much earlier, at 216 days, after initial vaccination. Again, a single vaccination was capable of mediating long term protection with one animal completely free of tumor up to 150 days post challenge (Figure 4). Staggering the 3rd dose of MS-OVA vaccination did not induce a substantially greater frequency of OVA-specific CD8^+^ T cells, however it facilitated maintenance of a greater proportion of CD62L^high^ (~45%), T_CM_ cells. This might explain why in this scenario, triply vaccinated mice had a longer median survival of 91 days compared to mice receiving one or two vaccinations at 46 days. Notably, even though greater proportions of CD62L^high^ OVA-specific CD8^+^ T cells were found in mice vaccinated twice (80%), the frequency of OVA-specific CD8^+^ T cells was three-fold lower. Overall, neither a high frequency of OVA-specific CD8^+^ T cells nor a high proportion of T_CM_ cells could solely confer prolonged tumor protection, reinforcing the idea that both quantity and quality are important in achieving long-term prophylactic immunity.

Attributing CD62L^high^ OVA-specific CD8^+^ T cells with a greater capacity to provide longer lasting tumor protection relates to their lymphoid migratory properties and proliferative potential when re-confronted with antigen in the context of tumor. Typically, a CD62L^high^ OVA-specific CD8^+^ T cell will reside in lymphoid compartments, interact with its cognate antigen on an APC and proliferate to generate effector CD8^+^ T cells to circulate and combat the tumor [41]. As such, it might be expected that a successful anti-tumor immune response will result in a high frequency of circulating OVA-specific CD8^+^ T cells after tumor challenge. Upon tumor challenge of MS-OVA vaccinated mice, the proliferation of OVA-specific CD8^+^ T cells does occur, however higher frequencies are reached in mice that eventually succumb to tumor burden (Figure 5). Interestingly, mice that survived tumor challenge did not regain a CD62L^high^ phenotype of CD8^+^ T cells in the blood faster than mice that succumbed to tumor burden. Large antigen depots associated with tumor growth could account for the continued CD8^+^ T cell proliferation and possibly exhaustion, but other tumor evasive mechanisms could also be at play. Tumor evasive properties are probably fine-tuned to be activated in the wake of a profound T_E_ response which may limit cytolytic functions. Possibly evasive mechanisms include down-regulation of MHC Class I that prevents T_E_ cells from engaging their tumor target [42], the recruitment of regulatory T cells that can directly and indirectly (modulation of cytokine environment) impairs T_E_ function [43] and the up-regulation of checkpoint ligands such as PD-L1 that are known to engage PD-1 on T_E_ cells and interfere with TCR signaling, impairing cytolytic function [44]. Overall, the results of this study highlights the complexity of a two-way cross-regulation between host immunity and a rapidly growing tumor.

Archaeosomes are capable of inducing anti-tumor CD8^+^ T cell responses even in the presence of tumor, however their usefulness in combating a tumor in both a prophylactic and therapeutic setting may be confounded by tumor induced immune evasive mechanisms. This is the case for most anti-tumor therapies and is why combinatorial approaches should be employed to simultaneously enhance functionality of vaccine induced CD8^+^ T cells while inhibiting regulatory mechanisms. A key advantage of archaeosomes is their ability to self-adjuvant vaccine responses. Indeed, many other particulate vaccines such as conventional liposomes, polymeric microspheres, nano-beads, virus-like particles, niosomes and proteosomes [45,46,47,48] require extraneous PAMP additives to adjuvant T cell responses.

While live vaccines derived from viral or bacterial vectors have thus far been the best choice for the induction of CD8^+^ T cell responses, particulate vaccines can offer some advantages, for example with homologous priming and boosting. Homologous vaccination with live vectors is often found to be ineffective as host immune responses target the vector and suppress subsequent boosts, as a result, heterologous prime and boost strategies have been the preferred method for inducing large numbers of CD8^+^ T cells with live vectors [49]. While these methods are thought to be logistically challenging due to difficulties in translating into the clinic and gaining GMP approval, they are showing promising results [34]. Additional attributes of archaeosomes include their ability to provide co-stimulatory signals without overt inflammation that can lead to the attenuation or deletion of T cells [22]. Liposomes have also been used with anchored targeting molecules directing them towards tumor sites to enhance or for drug delivery [50,51,52]. The combination of drug therapy with CD8^+^ T cell vaccines can have a synergistic affect when the immunogenicity of tumors can be enhanced, an example would be the administration of a chemotherapeutic such as drug 5-aza-2-deoxycitidine which increases tumor MHC class I expression, as well as some tumor antigens, enhancing survival in tumor bearing mice [53,54]. Other combinatorial anti-tumor therapies have been attempted by combining the pro-inflammatory GM-CSF with dendritic cell therapy, [55,56] or oncolytic viruses [57]. Since previous studies have shown that liposomes can enhance bioavailability of entrapped cytokines [58,59], the encapsulation of GM-CSF within archaeosomes with tumor antigen may be a promising approach that requires further investigation. Indeed archaeosomes are well poised for use in many combinatorial immunotherapy regimes.

## 5. Conclusions

Herein we have shown that archaeosomes constitute a robust particulate delivery system that can be used in a homologous repeat boost vaccination regime to induce high levels of OVA-CD8^+^ T cells reaching 45% of all circulating CD8^+^ T cells with three vaccinations. We demonstrated that further vaccinations skewed the response towards an effector memory phenotype and did not further increase the frequency. A late tumor challenge at seven months post initial vaccination showed that vaccinated animals were afforded protection compared to control. Therefore, archaeosomes constitute a robust particulate delivery system that can be used to unravel the kinetics of CD8^+^ T cell responses and memory maintenance and constitute an efficient vaccination regimen optimized for tumor protection.
